# Formulation of Broccoli Sprout Powder in Gastro-Resistant Capsules Protects against the Acidic pH of the Stomach In Vitro but Does Not Increase Isothiocyanate Bioavailability In Vivo

**DOI:** 10.3390/antiox8090359

**Published:** 2019-09-01

**Authors:** Masuma Zawari, Bettina Poller, Greg Walker, Andree Pearson, Mark Hampton, Anitra C. Carr

**Affiliations:** 1 Department of Pathology and Biomedical Science, University of Otago, Christchurch, P.O. Box 4345, Christchurch 8140, New Zealand; 2 School of Pharmacy, University of Otago, P.O. Box 56, Dunedin 9054, New Zealand

**Keywords:** isothiocyanates, broccoli sprout powder, enteric-coated capsules, gastro-resistant capsules, Nrf2

## Abstract

Broccoli sprout powder is a rich source of glucosinolates, which are hydrolysed to isothiocyanates in the presence of the enzyme myrosinase. We showed that in vitro incubation of broccoli sprout powder extract with isolated lymphocytes resulted in the upregulation of transcription factor Nrf2, however, there was no increase in Nrf2 protein levels in lymphocytes isolated 3 h following the ingestion of broccoli sprout powder by healthy volunteers. This highlights the general issue that potential health benefits of food-derived compounds can be compromised by limitations in bioavailability. In vitro experiments showed that the generation of isothiocyanates was reduced when the powder was first exposed to the low pH (1.2) of the stomach and then transferred to the higher pH (6.8) of the intestine. The loss of activity due to pre-exposure to the low stomach pH indicates that formulating the broccoli sprout powder in gastro-resistant formulations should increase that amount of isothiocyanate generated in the intestine for absorption. Gelatin capsules were hand-coated with either Eudragit^®^ L100 or Eudragit^®^ L100-55 and were assessed for their gastro-resistant properties using paracetamol as a model active for dissolution studies. Disintegration and dissolution studies showed that Eudragit^®^ L100-55 coated capsules and DRcaps^TM^ (Capsugel^®^) failed the United States Pharmacopeia (USP) requirements for gastro-resistant capsules, whereas the Eudragit^®^ L100 coated capsules passed. Five healthy participants were administered 1 g of broccoli sprout powder, ingested either with water or encapsulated in uncoated or gastro-resistant capsules. Urinary excretion of isothiocyanate metabolites over the 24 h period post ingestion was assessed by HPLC. Broccoli sprout powder and uncoated gelatin-encapsulated powder showed comparable excretion of isothiocyanate metabolites (18.4 ± 2.3 and 23.9 ± 2.7 µmol, respectively). The enteric coated capsules provided a significantly longer T_max_ than the uncoated gelatin capsules (15.4 ± 2.3 versus 3.7 ± 0.7 h, respectively), indicating protection from disintegration in the stomach, however, the excretion of isothiocyanate metabolites was significantly decreased compared with uncoated capsules (i.e., 8.5 ± 1.1 µmol). The lower in vivo formation or absorption of isothiocyanates observed for the gastro-resistant capsules may be due to participant variation in intestinal pH or transit times, resulting in inappropriate pH conditions or insufficient time for the complete disintegration and dissolution of the capsules.

## 1. Introduction

Isothiocyanates are a family of phytochemicals responsible for the potential health effects of cruciferous vegetables [1]. Disruption of plant cell structure results in the hydrolysis of the precursor glucosinolate by the enzyme myrosinase to generate isothiocyanates [2]. Isothiocyanates have a broad array of effects in biological systems, including the stimulation of cellular antioxidant systems by the activation of the Kelch-like ECH-associated protein 1 (Keap1) and nuclear factor erythroid 2 p45-related factor 2 (Nrf2) pathway [3,4]. Nrf2 is a transcription factor activated by chemicals, radiation and electrophiles, and can protect cells from oxidative stress [5]. The Nrf2 gene is expressed in most cell types and the protein is sequestrated in the cytoplasm by Keap1, which triggers the polyubiquitination of Nrf2 [6,7]. Ubiquitinated Nrf2 is rapidly degraded by the 26S proteasome, resulting in low levels of Nrf2 protein under basal state. The electrophilic properties of isothiocyanates result in the modification of the thiol groups of Keap1, causing dissociation of the two proteins and a resultant increase in intracellular Nrf2 levels. Nrf2, then, rapidly translocates into the nucleus, dimerizes with the small Maf protein and binds to its antioxidant responsive elements (ARE) in gene promoters of antioxidant enzymes, such as NAD(P)H quinone oxidoreductase 1 (NQO1), heme oxygenase 1 (HO-1), and modifier subunit for glutamate-cysteine ligase (GCLM) [5,7].

The upregulation of phase II antioxidant enzymes by sulforaphane, the major isothiocyanate from broccoli, has been reported in cell culture studies [8,9]. However, these effects are not always observed in human intervention studies [10,11], perhaps due to lower in vivo bioavailability of the isothiocyanates. Most intervention studies to date have been carried out using either fresh or cooked broccoli, or broccoli sprouts, which contain higher levels of glucosinolate than mature broccoli [12]. Different methods of processing the broccoli sprouts included freeze dried or air dried powder made from fresh product or a lyophilized extract made from sprouts boiled in water [13]. Myrosinase has been shown to be sensitive to heat inactivation and human studies have shown decreased isothiocyanate bioavailability of cooked versus raw broccoli [14,15,16], and lower bioavailability reported in dried broccoli sprout powder extract when heated versus fresh sprouts [17,18]. Several studies have reported that the consumption of broccoli sprout supplements devoid of an active myrosinase resulted in a lower generation of isothiocyanates [18,19].

The availability of enzyme cofactors and the pH of the reaction conditions can also affect formation of isothiocyanates [16,20,21,22]. Myrosinase in broccoli was shown to have the highest activity at pH 6.5–7 [23]. Ascorbic acid has been reported to act as a cofactor for myrosinase and higher enzyme activity was observed in the presence of ascorbic acid [24,25,26,27]. Furthermore, the formation of isothiocyanates is favored at neutral pH, while nitrile formation occurs under more acidic environments [28,29,30]. Therefore, the use of gastro-resistant formulations, which bypass the stomach and break down in the small intestine, may enhance the in vivo formation of isothiocyanates. There are a number of formulations available which are gastro-resistant. The simplest approach is to load the dry powder into a hard capsule and then coat it with an enteric polymer such as Eudragit^®^ L100. More recently, commercially available gastro-resistant capsules which do not require coating, as they are composed of an acid resistant formulation, e.g., DRcaps^TM^ (Capsugel^®^) have entered the market. One recent study investigated the bioavailability of broccoli sprout powder isothiocyanates using DRcaps^TM^ [31], however, no difference in bioavailability between DRcaps^TM^ and standard gelatin capsules was found. This could be due to these commercial capsules not meeting United States Pharmacopeia (USP) enteric dissolution requirements [32].

Therefore, the aim of this study was to develop and test gastro-resistant capsules that meet the USP dissolution and disintegration specifications for an enteric protected formulation. We hypothesised that enteric coating of the capsules would enhance the generation of isothiocyanates by delivering the powder to the small intestine, which has a slightly alkaline pH, more favourable conditions for isothiocyanate generation. The enteric coated capsules, which met the USP requirements, were assessed in a small randomised cross-over pilot study to determine the impact of stomach exposure on broccoli sprout powder isothiocyanate generation in vivo. Since isothiocyanates have a bitter taste and a pungent odour upon formation in the mouth, encapsulation of the broccoli sprout powder within capsules also masks its flavour and taste, thus improving the palatability of the product.

## 2. Materials and Methods

### 2.1. Reagents

HPLC grade methanol was from Thermo Fisher Scientific (Sunnyvale, CA, USA). Acetonitrile was from Global Science (Auckland, New Zealand). Potassium phosphate dibasic, potassium phosphate monobasic, Tween 80 and hydrochloric acid (37%) were from Merck KGaA (Darmstadt, Germany). Sodium phosphate tribasic dodecahydrate, acetaminophen and benzene-1,2-dithiol were from Sigma-Aldrich (St Louis, MO, USA). Polyethylene glycol 400 were from BDH laboratory reagents (Poole, England). DRcaps^TM^ were purchased from Capsugel^®^ (Morristown, NJ, USA), Eudragit^®^ L100 and Eudragit^®^ L100-55 powder was purchased from Evonik Australia Industries (Melbourne, Victoria, Australia), propylene glycol BP was purchased from PSM Health care (Auckland, New Zealand), and the hard gelatin capsules (size 0) were obtained from NZ Nutritionals^®^ (Christchurch, New Zealand). All the other reagents and solvents were purchased from Sigma Aldrich (Auckland, New Zealand).

### 2.2. Preparation of Broccoli Sprout Powder

Broccoli sprout powder was supplied by Claridges Organic Ltd. (Christchurch, New Zealand). New Zealand broccoli seed was sprouted for five days using a rotary drum sprouting machine (commercial size). Once sprouted, the five-day-old sprouts were washed to rid the sample of ungerminated seed and seed husks. The wet sprouts were spun dried, frozen, chopped and put into a freeze drier. A 24-h cycle in the freeze drier totally dehydrated the sprouts to below 4% moisture. The dried sprouts were then milled to the required particle size to fill capsules. Microbial testing of the sprout powder was carried out by Eurofins NZ Laboratory Services Ltd., an International Accreditation New Zealand (IANZ) accredited laboratory.

### 2.3. Lymphocyte Isolation and Treatment with Broccoli Sprout Powder Extract

Blood was collected from healthy donors (NZ Human and Disability Ethics Committee approval number URA0612083) and density gradient centrifugation was used to separate the peripheral blood mononuclear cells (PBMCs). Lymphocytes were then purified using magnetic antibody-coated beads that selectively removed monocytes (CD14+), thrombocytes and erythrocytes (CD235a+ and CD61+ cells, respectively) [33]. The remaining lymphocytes (92 ± 8 % purity) were counted using a hemocytometer and the average number of cells was 32 ± 5 × 10^6^ from 50 mL of blood. Lymphocytes (5 × 10^6^ cells) were treated for 2 h with broccoli sprout powder extract containing known concentrations of isothiocyanates (1–5 µmol/L). The cells were incubated in complete RPMI 1640 medium supplemented with 10% heat-inactivated fetal bovine serum, penicillin (100 units/mL) and streptomycin (100 µg/mL) at 37 °C with 5% CO_2_. The broccoli sprout powder extract was made with 0.2 M sodium phosphate tribasic dodecahydrate buffer (pH 6.8) and the isothiocyanate concentration was quantified by HPLC, as described below.

### 2.4. Nrf2 Immunoblotting

For Western blots, lymphocytes were lysed by incubation with sample loading buffer (138 mM Tris pH 6.8, 2% SDS, 0.9 M sucrose and 10% β-mercaptoethanol) and proteins were resolved by SDS-PAGE using 8%–16% resolving gels. Proteins resolved by SDS-PAGE were electrophoretically transferred to polyvinylidene difluoride membrane using Bio-Rad Mini Trans-Blot apparatus (Bio-Rad Laboratories, Hercules, CA, USA). Non-specific antibody binding sites were blocked by incubating membranes for 1 h at room temperature with 5% (*w*/*v*) non-fat dried milk, dissolved in Tris-buffered saline (20 mM Tris-HCl, 140 mM NaCl, pH 7.6) containing 0.05% (*v*/*v*) Tween-20 (TBST). Incubation with primary antibodies was performed overnight at 4 °C in TBST containing 3% (*w*/*v*) non-fat dried milk. Membranes were thoroughly washed with TBST, then incubated with an appropriate horseradish peroxidase-conjugated secondary antibody for 1 h at room temperature. Membranes were probed with antibodies specific for Nrf2 (Abcam, Cambridge, UK) and β-actin (Sigma-Aldrich, St. Louis, MO, USA) was used as the loading control. The blots were developed using an enhanced chemiluminescence kit (GE Healthcare/Amersham Biosciences, Little Chalfont, UK) and band intensities were measured using Q9 Alliance software (Cambridge, UK). The densitometry for Nrf2 bands were normalised to the loading control β-actin (Nrf2/β-actin). Fold changes in Nrf2 level in the treated samples were then calculated relative to the untreated sample.

### 2.5. Preparation of Gastro-Resistant Capsules

#### 2.5.1. Capsule Filling

Gelatin capsules, size 0, were filled with broccoli sprout powder using a hand-held capsule filling system (ProFiller^®^1000, Torpac, Fairfield, NJ, USA) under clean conditions. Capsules (*n* = 10) were weighed individually, emptied, and the shells were weighed to determine the net mass of their contents. Weight variation of content was determined as described in the USP (General Chapter: <905> Uniformity of Dosage Units specification).

#### 2.5.2. Capsule Coating

The enteric-coating solutions were prepared by dissolving either 58 g of Eudragit^®^ L100 in 223 mL ethanol or 18 g Eudragit^®^ L100-55 in a solvent mixture of 144 mL isopropanol, 76 mL acetone and 10 mL deionised water. The prepared polymer solutions were shaken at 290 rpm (Orbital mixer incubator, Ratek Instruments Pty Ltd., Boronia, Australia) for 15–20 h, followed by sonication in the water bath for 2 h. Plasticizer, 5.8 g, in propylene glycol was added slowly to the Eudragit ^®^ L100 solution with stirring, 2.8 g polyethylene glycol 400 and 0.2 g Tween 80 were added to the Eudragit^®^ L100-55 solution. After addition of the propylene glycol, the solution was stirred for 10 min. The coating solutions were stored in the dark at room temperature overnight for aging. The next day, capsules were coated using the capsule dip coater (ProCoater^®^, Torpac, Fairfield, NJ, USA), following the recommended procedure of the company. After the first coat, the capsules were left at room temperature for 1 h, after which a second coat was applied. The coated capsules were dried at 25 ± 3 °C and 55 ± 3% relative humidity for 3 days, after which they were stored at room temperature in a closed container.

### 2.6. Evaluation of Gastro-Resistant Capsules

#### 2.6.1. Disintegration Testing

Disintegration was performed using the apparatus COPLEY disintegration tester DTG 2000 (Nottingham, UK). The coated capsules were placed in a basket-rack assembly consisting of three open-ended transparent tubes, which was subsequently transferred to a vessel containing simulated gastric fluid (0.1 M HCl). The disintegration apparatus was run for 1 h at 37 °C. To simulate intestinal fluid, 250 mL of 0.2 M sodium phosphate dodecahydrate was added to 750 mL of the 0.1 M HCl to adjust to a pH of 7.4 and discs were added to the transparent tubes. The apparatus operated in the simulated intestinal fluid for another hour at 37 °C.

#### 2.6.2. Dissolution Testing

Paracetamol (450 ± 13 mg) was filled into size zero capsules, which were then coated as described previously. The dissolution test (*n* = 3) was performed according to the USP (2011) basket method (Apparatus 1; USP <711> Dissolution) using a ZRS-8 dissolution tester (Tianjin Tianda Tianfa Technology, Tianjin, China). One coated capsule filled with paracetamol was placed into a basket. The basket was rotated at a speed of 50 rpm in 750 mL of simulated gastric fluid (0.1 M HCl) at 37 °C. After 2 h, either 250 mL of 0.2 M sodium phosphate dodecahydrate was added to the previous media to adjust it to a pH of 7.4 or simulated gastric fluid was replaced with 1000 mL of a buffer solution containing 22.4 mM sodium hydroxide and 50 mM potassium dihydrogen phosphate to elevate the pH to 6.8 and maintained at 37 °C. The rotation speed of the basket was changed to 100 rpm. At certain time points, a 1-mL sample was withdrawn and analysed at 249 nm using a UV-VIS spectrophotometer (Ultraspec 2000, Pharmacia Biotech, Piscataway, NJ, USA).

### 2.7. Pilot Study of Gastro-Resistant Capsules

Five healthy adult participants (four males and one female, between the ages of 40 and 50 years) were recruited and provided written informed consent. The study was approved by the University of Otago Human Research Ethics Committee (reference number H15/113). Participants were asked to refrain from consuming cruciferous vegetables (e.g., broccoli, mustard, horseradish, wasabi) that contain isothiocyanates or glucosinolates for 24 h before and during the study. The pilot study comprised a randomised cross-over design and participants consumed 1 g broccoli sprout powder mixed with water as control or delivered in enteric-coated and non-coated gelatin capsules (total of three capsules each). Participants did not fast before each intervention. The participants provided a pre-dosing urine sample and the entire urine excreted during the 24 h post ingestion was collected every time they visited the bathroom. Urine samples were stored at 4 °C overnight or at −20 °C if stored for longer (48 h) until analysed. There was a 24-h washout period between each intervention to ensure that all isothiocyanate metabolites were cleared from the circulatory system. Peripheral blood samples were also collected pre-dosing and 3 h post-dosing for some participants. The total isothiocyanate metabolites in the plasma and urine samples was determined using a cyclocondensation assay and HPLC with UV detection, as described below [34].

### 2.8. Isothiocyanate Derivatisation and Detection by HPLC

Plasma proteins were precipitated by adding 6% polyethylene glycol (MW 8000) to 500 μL aliquots of plasma. The samples were kept on ice for 10 min and then centrifuged at 12,000× *g* for 5 min at 4 °C. Aliquots of the deproteinated plasma and the urine samples were diluted with 100 mM potassium phosphate buffer, pH 8.5 before the addition of 20 mM benzene-1,2-dithiol. The cyclocondensation assay was carried out at 65 °C for 1 h. The cyclocondensation product of the isothiocyanates and their metabolites (dithiocarbamates) was analysed by HPLC with UV detection, as described previously [35]. Briefly, the cyclocondensation product was analysed using an Alliance HPLC system with a Waters 996 Photo Diode Array detector (365 nm). The samples were run for 10 to 20 min and were eluted using an isocratic method, with the mobile phase comprising 80% methanol and 20% water using a synergi 2.5 µm, Hydro-RP 100 × 3 mm, C18 column (Phenomenex Inc, San Jose, CA, USA). The standard was synthesised by reacting known concentrations of pure sulforaphane with 20 mM benzene-1,2-dithiol at 65 °C for 1 h. Chromeleon software (Dionex/Thermo Fisher Scientific, Sunnyvale, CA, USA) was used for data acquisition and calculating the peak area.

### 2.9. Ascorbic Acid Content of Broccoli Sprout Powder

Broccoli sprout powder (10 mg) was extracted with 400 µL 0·54 M perchloric acid containing the metal chelator diethylene triamine pentaacetic acid (DTPA) (100 µmol/L), followed by centrifugation. The supernatant was analysed by HPLC with electrochemical detection, as described previously [36].

### 2.10. Statistical Analyses

Data are presented as mean ± SEM for comparisons between group means. Analysis of paired data was determined using two-tailed Students *t*-test with *p* ≤ 0.05 reported as significant. One-way analysis of variance and post-hoc multiple comparisons tests were carried out using GraphPad Prism 7 (GraphPad Software, La Jolla, CA, USA).

## 3. Results

### 3.1. Nrf2 Induction in Lymphocytes Exposed to Broccoli Sprout Powder Extract In Vitro and In Vivo

**Figure 1:**
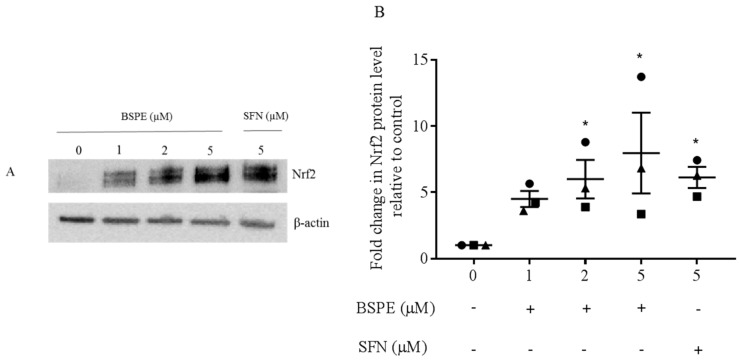
Nrf2 protein levels after treatment of lymphocytes with broccoli sprout powder extract. (**A**) Lymphocytes were purified from peripheral blood and treated with broccoli sprout powder extract (BSPE) or pure sulforaphane (SFN) for 2 h (representative western blot from 3 individual experiments). (**B**) Densitometry analysis of Nrf2 protein band relative to control and β-actin level. Results are mean ± SEM (* *p* ≤ 0.05, ANOVA with Holm-Sidak’s multiple comparisons test).

Purified lymphocytes were treated for 2 h with broccoli sprout powder extract (which had been quantified for in vitro isothiocyanate formation) and Nrf2 protein levels were measured by gel electrophoresis and western immunoblotting (Figure 1). There was a dose-dependent increase in Nrf2 protein levels, which was comparable to an equivalent concentration of pure sulforaphane, the major isothiocyanate in broccoli. Analysis of Nrf2 induction was also carried out in lymphocytes isolated from three individuals 3 h after ingestion of 2.5 g broccoli sprout powder made up in water or delivered using standard gelatin capsules. There were, however, no changes detected in Nrf2 protein levels in lymphocytes isolated 3 h after ingestion of the broccoli sprout powder (data not shown). The plasma isothiocyanate metabolite levels measured 3 h post-supplementation ranged between 0.1 and 1.4 µM. These results suggest the need for formulations to enhance isothiocyanate levels in vivo in order to optimise the biological activity of broccoli sprout powder.

### 3.2. pH-Dependence of Isothiocyanate Formation from Broccoli Sprout Powder

**Figure 2:**
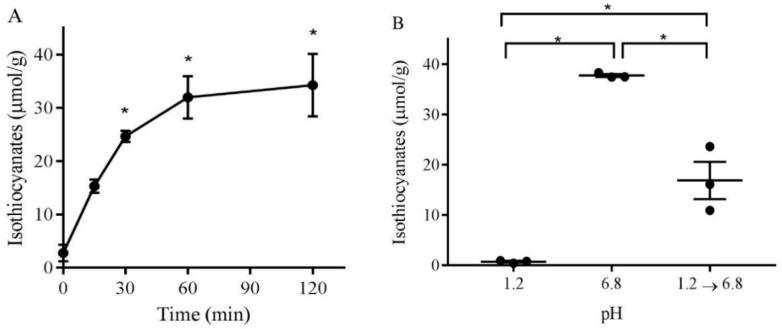
Generation of isothiocyanates from broccoli sprout powder under neutral and acidic pH. (**A**) Broccoli sprout powder was incubated with pH 6.8 buffer at 37 °C. Aliquots were removed at the indicated time points, derivatised using the cyclocondensation assay, and quantified using HPLC with UV detection. Significant formation was detected at 0.5–2 h (*p* ≤ 0.05, one way repeated-measures analysis of variance with Dunnett’s multiple comparison test). (**B**) Broccoli sprout powder was mixed with the required solutions (pH 1.2 or pH 6.8) for 1 h. The incubation of broccoli sprout powder at pH 1.2 for 1 h then changing the pH to 6.8 by adding 0.2 M phosphate buffer (pH 1.2 → pH 6.8) for another hour showed a significant reduction of isothiocyanates. Results are mean ± SEM, *n* = 3 (* *p* ≤ 0.05, two-tailed Students *t*-test).

The incubation of broccoli sprout powder in buffer (pH 6.8 at 37 °C) resulted in the formation of isothiocyanates, the kinetics of which are shown in Figure 2A. Heat inactivation of the enzyme by boiling the sprout solution or heating the sprout powder to 100 °C decreased the generation of isothiocyanates by >90% (3.4 ± 0.4 versus 37.9 ± 2.3 µmol/g, *n* = 3). To investigate the effect of the pH environment of the stomach and small intestine on isothiocyanate formation, the powder was incubated at pH 1.2 and 6.8, respectively. After 1 h of incubation at 37 °C, isothiocyanates were measured (Figure 2B). At pH 1.2, there was negligible generation of isothiocyanates compared to at pH 6.8 (0.7 ± 0.1 and 37.7 ± 0.2 µmol/g, respectively). To mimic the impact that the low pH of the stomach may have had on the subsequent generation of isothiocyanates in the small intestine, the powder was incubated at pH 1.2 for 1 h, then the pH was elevated to pH 6.8 for another hour and then assayed for isothiocyanate formation. The transition from pH 1.2 to 6.8 resulted in the intermediate formation of isothiocyanates (16.9 ± 3.0 µmol/g). This amount of isothiocyanate generation was significantly lower, representing 44% of the isothiocyanate generated at pH 6.8 exposure only. These in vitro experiments demonstrated that the formation of broccoli sprout powder active ingredients was influenced by the lower pH of the stomach and therefore would likely benefit from being formulated into a gastro-resistant capsule.

Since ascorbate is required for optimal myrosinase activity, the effect of additional ascorbate on in vitro isothiocyanate generation was assessed. The ascorbate content of the broccoli sprout powder extract was 156 µmol/L (equivalent to 6.2 µmol/g powder) and the addition of a range of ascorbate concentrations (300–2000 µmol/L) did not enhance the formation of isothiocyanates (data not shown). This suggests that there was already sufficient ascorbate present in the powder for optimal myrosinase activity and that the addition of ascorbic acid to capsules was not required.

### 3.3. Formulation and Testing of Gastro-Resistant Capsules

To make gastro-resistant gelatin capsules, they were coated with either Eudragit^®^ L100 polymer or Eudragit^®^ L100-55 polymer. Eudragit^®^ L100 is triggered at pH 6–7 and is designed to break down in the small intestine, and Eudragit^®^ L100-55 is triggered at pH 5.5 and is targeted for the duodenum. To determine whether the enteric coating method met the specifications for a gastro-resistant capsule, these capsules were loaded with paracetamol as a model drug that can be easily quantified at 249 nm. In vitro dissolution studies for paracetamol release from enteric coated and non-coated capsules was determined in simulated gastric fluid (pH 1.2) and simulated intestinal fluid (pH 6.8 and pH 7.4). Uncoated gelatin capsules and commercial DRcaps^TM^ were also filled with paracetamol and evaluated for dissolution properties.

**Figure 3:**
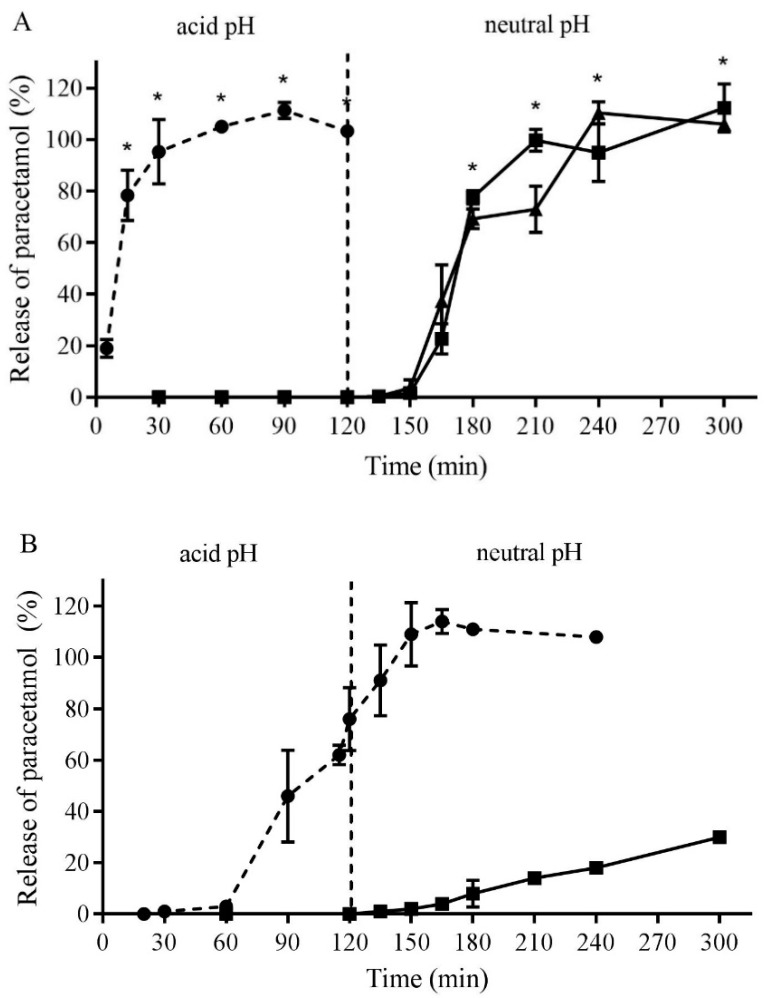
In vitro dissolution of Eudragit^®^ L100 and Eudragit^®^ L100-55 coated and non-coated capsules. (**A**) Paracetamol release from standard gelatin capsules (●, dashed line) or enteric coated capsules (◼ pH 7.4, ▲ pH 6.8) in simulated gastric fluid (acid pH) for 120 min, followed in simulated intestinal fluid (neutral pH) for 180 min. (**B**) Paracetamol release from uncoated DRcaps^TM^ (●, dashed line) or DRcaps^TM^ coated with Eudragit^®^ L100-55 (◼ pH 6.8). Results are mean ± SEM, *n* = 3 (* *p* ≤ 0.05, ANOVA with Dunnett’s multiple comparisons).

The dissolution profile for the Eudragit^®^ L100-coated capsules showed that no paracetamol was released over the first 2 h in 0.1 M HCl (pH 1.0) (Figure 3A). After the pH was changed to either 6.8 or 7.4, almost 80% of the drug was released within 60 min and 100% by 90 min. The capsules did not show any signs of disintegration, cracking or softening after 1 h in the simulated gastric fluid and completely dissolved in the simulated intestinal fluid within 45 min [37]. In contrast, the non-coated gelatin capsules released 80% of the paracetamol at 15 min in the simulated gastric juice (Figure 3A). However, gelatin capsules coated with Eudragit^®^ L100-55 polymer dissolved within 120 min in 0.1 M HCl (pH 1.0), therefore failed the USP dissolution test for enteric coated capsules (data not shown). Uncoated DRcaps^TM^ also failed the USP dissolution test, with nearly 80% release of paracetamol at acid pH, whereas DRcaps^TM^ coated with Eudragit^®^ L100-55 released only 30% of the paracetamol after 180 min at pH 6.8 (Figure 3B). Overall, these in vitro results demonstrated that coating gelatin capsules with Eudragit^®^ L100 polymer would be suitable for delayed release delivery to the small intestine, thus protecting the broccoli sprout powder from exposure to the acid environment of the stomach.

### 3.4. In Vivo Pharmacokinetic Study

**Figure 4:**
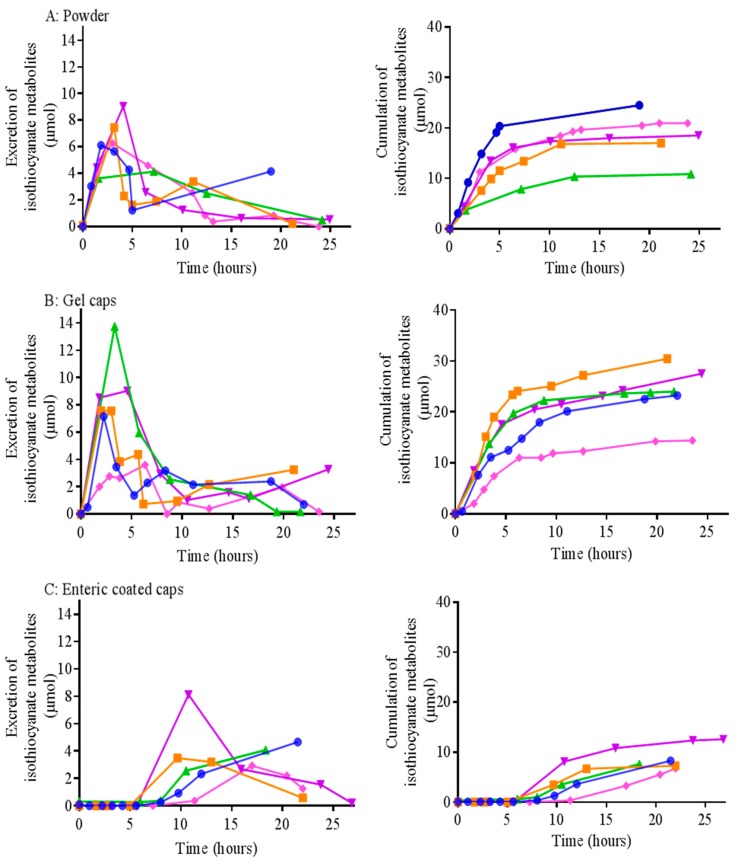
Encapsulation of broccoli sprout powder and the effect on urinary excretion of isothiocyanate metabolites. Five healthy individuals ingested 1 g of broccoli sprout powder delivered using water (**A**), standard gelatin capsules (**B**) or Eudragit^®^ L100 polymer coated capsules (**C**) containing the equivalent of 1 g powder per three capsules. Urine samples were collected at baseline and over 24 h after ingestion of broccoli sprout powder. Samples were processed by the cyclocondensation assay and analysed by HPLC with UV detection. Pharmacokinetic data are shown on the left hand and corresponding cumulative data are shown on the right hand. No significant differences were detected between normal capsules and broccoli sprout powder delivered in water (*p* = 0.224; one way repeated-measures ANOVA with Tukey’s multiple comparisons test). Enteric coated capsules showed a delay in the peak, but significantly lower isothiocyanate metabolites compared to the powder or normal capsules (*p* = 0.029, 0.003 respectively; one way repeated-measures ANOVA with Tukey’s multiple comparisons tests). Different participants are indicated by different coloured lines and symbols (*n* = 5).

In vivo generation of isothiocyanates from broccoli sprout powder was analysed in five healthy individuals by measuring the excretion of metabolites. Although standard gelatin capsules showed slightly higher isothiocyanate formation compared to broccoli sprout powder delivered using water (Figure 4), this was not statistically significant. The peak maximum appeared approximately 3.7–3.9 h post ingestion of standard gelatin capsules or broccoli sprout powder delivered in water, respectively (Table 1), and by 12 h, almost all of the isothiocyanate metabolites were eliminated (Figure 4). Some individuals showed the appearance of a second smaller peak at later time points, which may indicate microbiome-mediated metabolism.

Enteric-coated capsules showed a delay (average time of 15 h) in the urinary excretion of isothiocyanate metabolites (Figure 4 and Table 1), indicating the passage of the capsule through the acidic conditions of the stomach. The collection of urine over 48 h did not show any additional excretion of isothiocyanate metabolites (data not shown). The mean absolute amount of isothiocyanate metabolites excreted was 18.4 ± 2.3, 23.9 ± 2.7 and 8.5 ± 1.1 µmol for broccoli sprout powder, standard gelatin capsules and enteric coated capsules, respectively. There was no significant difference between the broccoli sprout powder and the standard gelatin capsule (*p* = 0.224). This data indicates that the dissolution of the enteric coated capsules in the intestine may not be complete during transit through the small intestine.

## 4. Discussion

We carried out this study to determine if the in vivo formation of broccoli sprout isothiocyanates could be improved through enteric encapsulation. We showed that in vitro treatment of isolated lymphocytes with broccoli sprout powder extract provided a clear induction of Nrf2 protein levels, comparable to induction by purified sulforaphane. However, no upregulation of Nrf2 was detected in lymphocytes isolated from volunteers 3 h after the ingestion of broccoli sprout powder. Plasma concentrations of the isothiocyanates (~1 µM) might not be high enough to induce significant Nrf2 activation, or the difference may be due to the isothiocyanates being present as conjugates with glutathione. These dithiocarbamates are reversible and as such, retain biological activity [38], however, higher levels may be necessary to activate Nrf2 in vivo. This highlights the general issue that potential health benefits of compounds derived from foodstuffs can be compromised by limitations in bioavailability. Improved formulation may help to optimise isothiocyanate uptake from broccoli sprout powder.

Our in vitro data confirmed that the formation of isothiocyanates under acidic conditions mimicking the stomach (pH 1.2) was minimal compared to the neutral conditions expected in the intestines (pH 6.8) [23,39,40]. This could be due to the lower activity of myrosinase at acidic pH, and/or the formation of alternative products, such as nitriles, which occurs at pH ≤ 4 [41,42]. The recovery of a proportion (47%) of the in vitro isothiocyanates generation following an increase in pH (from 1.2 to 6.8) indicates that the enzyme was not fully inactivated by the initial low pH. It is well known that the presence of active myrosinase is required for optimal isothiocyanate formation, as evidenced by decreased isothiocyanate levels in the presence of heat-inactivated myrosinase [31], which we also observed in our study. Although the addition of ascorbate (300–750 µmol/L) to fresh broccoli sprout or broccoli sprout powder has been reported to increase myrosinase activity [25,27], we did not observe any increase with the addition of ≥300 µmol/L at pH 6.8. This indicates that the ascorbate content of the broccoli sprout powder (156 µmol/L) was sufficient for optimal activity of the myrosinase.

When broccoli sprout powder was delivered in vivo using water or in standard gelatin capsules, which disintegrate rapidly, there was no significant difference in isothiocyanate metabolites detected in urine. Delivering broccoli sprout powder using water or standard gelatin capsules showed early urinary excretion of isothiocyanate metabolites (T_max_ < 4 h), suggesting rapid in vivo absorption. By 24 h, levels had returned to baseline, which has been reported in other studies [13,15,16]. A second smaller peak appeared in some of the individuals at a later time point, which might be due to the myrosinase activity associated with gastrointestinal microflora. Bacteria associated with gastrointestinal microflora have myrosinase activity [43,44], however bacterial myrosinase activities are less efficient, giving only 10%–20% bioavailability [16,44].

To attempt to improve the in vivo formation of broccoli sprout powder isothiocyanates by delivering them to the neutral pH conditions of the small intestine, we coated standard gelatin capsules and DRcaps^TM^ with the pH-sensitive polymers Eudragit^®^ L100 (triggered at pH 6–7 and targeted to the small intestine), and Eudragit^®^ L100-55 (triggered at pH 5.5 and targeted to the duodenum). Using paracetamol as a model drug, disintegration and dissolution studies confirmed that the coating technology using the synthetic polymer Eudragit^®^ L100 was suitably robust for enteric protection. However, we observed a significant reduction in isothiocyanate generation with the enteric coated capsules in the in vivo study. We are aware of one other study that has investigated the in vivo formation of isothiocyanates using commercially available pH sensitive capsules [31]. These investigators reported comparable urinary excretion of isothiocyanate metabolites between standard gelatin capsules and the pH-sensitive DRcaps^TM^ [31]. However, we showed that the commercially available DRcaps^TM^ used in that study did not meet USP disintegration or dissolution criteria, hence may not deliver the capsule contents to the desired location.

The longer T_max_ observed with the Eudragit^®^ L100 coated capsules relative to the standard gelatin capsules (>15 h vs. <4 h, respectively) indicates that they did indeed pass through the stomach and into the intestines prior to dissolution. The lower isothiocyanate formation using enteric coated capsules may be due to a combination of factors, such as the slow formation of isothiocyanates by myrosinase (as indicated by the in vitro kinetic data), the complete in vitro release of the capsule contents taking at least 90 min, and the large variability in intestinal pH [45]. Thus, the capsules may not have had sufficient time to break down during transit through the small intestine [46]. The mean transit time of the small intestine has been reported to be approximately 84 min, but there is large variability between individuals (from as short as 15 min to as long as 5 h) [47]. Variation depends on various factors, such as gastrointestinal motility, the quality and quantity of food ingested, dietary fiber content, mobility, stress, disease and drugs [48]. Although, the mean transit time can also vary with age [49], our participants were of a similar age group. Isothiocyanate bioavailability can be modulated by food structure and composition and in some studies, subjects have been asked to fast prior to intervention [11,18,50]. In this study, the participants were not asked to fast prior to ingesting the sprout powder or capsules. The experiments were undertaken early on a working day, with participants reporting the consumption of a light breakfast before arriving at the study site. Despite the variable nature of food intake, all the participants had considerably lower isothiocyanate bioavailability with the Eudragit^®^ L100 enteric-coated capsules, indicating that gastric emptying of the preparation was not a variable limiting bioavailability.

The low bioavailability for the enteric-coated formulation maybe due to the capsule not releasing the broccoli sprout powder contents rapidly and completely enough during the transit time in the small intestine [51]. Enteric-coated gelatin capsules were used in this study as they do not require specialist equipment and are often used in the early stages of development for assessing the gastric protection of acid sensitive agents. To facilitate more efficient pH-triggered release of the broccoli sprout powder at the small intestine, novel dosage forms should be investigated. For example, the multiple unit pellet system (MUPS) containing enteric-coated pellets has been shown to have superior bioavailability than conventional dosage forms, such as tablets and capsules [52,53]. The improved bioavailability of MUPS has been attributed to the small size of the enteric coated pellets, which increases interaction with the tract surface for drug absorption and allows faster drug dissolution at the higher pH of the small intestine.

## 5. Conclusions

In vitro testing of our enteric coated capsules ensured that they met USP criteria. We were able to delay the dissolution of the enteric coated capsules in vivo, however, there was lower isothiocyanate formation. This was possibly due to a combination of factors, such as the slow release of the capsule contents, the slow generation of the isothiocyanates by myrosinase, and the variability in participant intestinal pH or transit times, resulting in inappropriate pH conditions or insufficient time for a complete disintegration and dissolution of the capsules. Alternatively, the intestines may not be an appropriate target for optimal isothiocyanate formation or absorption.

## Figures and Tables

**Table 1:** Urinary excretion of isothiocyanate metabolites after administration of one gram of broccoli sprout powder in water or delivered in normal gelatin capsules or enteric coated capsules.

	T_max_ (h)			C_max_ (µmol)			Total Isothiocyanate Metabolites (µmol)		
Subject No.	Powder	Gel-Cap	Enteric-Cap	Powder	Gel-Cap	Enteric-Cap	Powder	Gel-Cap	Enteric-Cap
1	1.8	2.3	21.5	6.1	7.2	4.7	24.5	23.2	8.3
2	3.2	2.0	9.7	7.5	7.6	3.5	17.0	30.5	7.3
3	7.2	3.3	18.3	4.1	13.8	4.1	10.9	24.0	7.6
4	4.1	4.6	10.8	9.1	9.0	8.1	18.5	27.5	12.6
5	3.0	6.3	17.0	6.3	3.6	2.9	20.9	14.4	6.8
mean	3.9	3.7	15.4 *	6.6	8.2	4.7	18.4	23.9	8.5 *
SEM	0.9	0.7	2.3	0.8	1.6	0.9	2.3	2.7	1.0

The time (T_max_) when maximum concentration (C_max_) of isothiocyanate metabolites was observed. * *p* ≤ 0.05 relative to normal gelatin capsules and broccoli sprout powder by one-way ANOVA with Tukey’s multiple comparisons test.
